# Hydrogen peroxide stimulates nuclear import of the POU homeodomain protein Oct-1 and its repressive effect on the expression of Cdx-2

**DOI:** 10.1186/1471-2121-11-56

**Published:** 2010-07-16

**Authors:** Peixiang Wang, Tianru Jin

**Affiliations:** 1 Div. of Cell and Molecular Biology, Toronto General Research Institute, University Health Network, 10-354 Toronto Medical Discovery Tower, The MaRS Building, 101 College St., Toronto, Ontario, M5G 1L7, Canada; 2 Dept. of Laboratory Medicine and Pathobiology, University of Toronto, 1 King's College Circle, Toronto, Ontario, M5 S 1A8, Canada; 3 Dept. of Medicine, University of Toronto, 200 Elizabeth Street, Toronto, Ontario, M5G 2C4, Canada; 4 Department of Physiology, University of Toronto, 1 King's College Circle, Toronto, Ontario, M5 S 1A8, Canada; 5 Dept. of Nutrition, School of Public Health, Sun Yat-Sen University, 74 2nd Zhongshan Road, Guangzhou, Guangdong Province, 510080, PR China

## Abstract

**Background:**

The ubiquitously expressed POU homeodomain protein Oct-1 serves as a sensor for stress induced by irradiation. We found recently that in pancreatic and intestinal endocrine cells, Oct-1 also functions as a sensor for cyclic AMP (cAMP). The caudal homeobox gene Cdx-2 is a transactivator of proglucagon (gcg) and pro-insulin genes. Oct-1 binds to Cdx-2 promoter and represses its expression. cAMP elevation leads to increased nuclear exclusion of Oct-1, associated with reduced recruitment of nuclear co-repressors to the Cdx-2 promoter and increased Cdx-2 expression.

**Results:**

We show in this study that inducing oxidative stress by hydrogen peroxide (H_2_O_2_) increased nuclear Oct-1 content in both pancreatic α and β cell lines, as well as in a battery of other cells. This increase was then attributed to accelerated nuclear import of Oct-1, assessed by Fluorescence Recovery After Photobleaching (FRAP) using green fluorescence protein (EGFP) tagged Oct-1 molecule. H_2_O_2 _treatment was then shown to stimulate the activities of DNA-dependent protein kinase (DNA-PK) and c-jun N-terminal kinase (JNK). Finally, increased Oct-1 nuclear content upon H_2_O_2 _treatment in a pancreatic α cell line was associated with reduced Cdx-2 and gcg mRNA expression.

**Conclusion:**

These observations suggest that Oct-1 functions as a sensor for both metabolic and stress/survival signaling pathways via altering its nuclear-cytoplasmic shuttling.

## Background

A transcription factor may serve as a sensor for different signaling pathways via altering gene expression profiles. For example, members of Foxo protein family were shown to mediate stress signaling via promoting its nuclear translocation and Foxo pathway downstream target gene expression, while insulin and insulin-like growth factor-1 (IGF-1) can block this pathway via stimulating Foxo protein phosphorylation at certain Ser/Thr residues, followed by its nuclear exclusion and degradation [1,2].

Oct-1 is a member of the POU domain transcription factor [3,4]. The protein in this family typically contains a bipartite DNA binding domain, in which two sub-domains are covalently connected by a flexible linker. These two sub-domains normally recognize DNA through major groove interaction on the opposite sides of the helix. The classical recognition sequence is known as the octamer motif "ATGCWAAT", where W can be either "A" or "T". This ubiquitously expressed transcription factor exerts multiple biological functions via up- or down-regulating the expression of a large profile of target genes in different cell lineages [5-8]. Recent studies indicated that Oct-1 functions as a sensor for radiation mediated stress via enhanced phosphorylation at multiple Ser/Thr sites in the N terminus of the molecule by DNA-dependent protein kinase (DNA-PK) [9-11]. Lately, we reported that Oct-1 binds to the promoter region of Cdx-2, a homeobox gene expressed in pancreatic islets and intestinal endocrine L cells, via the typical ATGCTAAT motif. We observed that nuclear content of Oct-1 can be reduced in response to cyclic AMP (cAMP) elevation in pancreatic and intestinal endocrine cells, and this reduction is associated with increased expression of Cdx-2 and its downstream target gene, the proglucagon gene (gcg). Furthermore, cAMP elevation reduced the binding of Oct-1 to Cdx-2 promoter and the recruitment of nuclear co-repressors, including silencing mediator of retinoid and thyroid hormone receptors (SMRT) and histone deacetylase 1 (HDAC1)[12]. These observations suggest that Oct-1 functions as a transcriptional repressor for a set of target genes, while cAMP elevation in response to the stimulation by peptide hormones leads to the release of the repressive effect.

In this study, we assessed the effect of hydrogen peroxide (H_2_O_2_) on Oct-1 cytoplasmic-nucleus shuttling. H_2_O_2 _treatment in pancreatic glucagon and insulin producing cell lines, as well as a battery of other cell lines and primary smooth muscle cells, was shown to increase nuclear Oct-1 content and Oct-1 nuclear translocation. In the Cdx-2 and Gcg expressing pancreatic islet α cell line, this was associated with increased c-jun N-terminal kinase (JNK) activation and DNA-PK activity, and decreased Cdx-2 and gcg mRNA expression. We suggest that Oct-1 exerts an important role in metabolic homeostasis by functioning as a sensor not only for cAMP, but also for oxidative stress.

## Results

### H_2_O_2 _treatment increases nuclear Oct-1 levels

**Figure 1:**
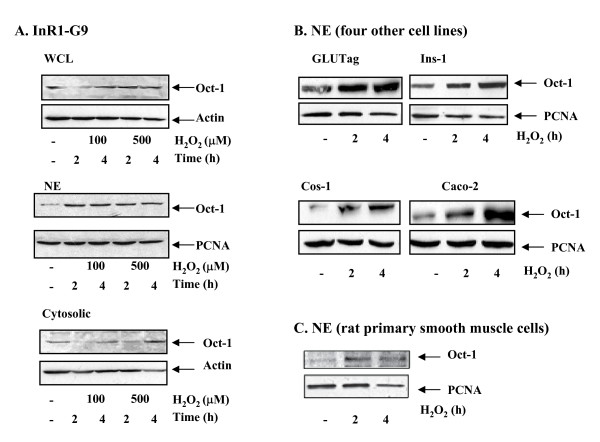
**H_2_O_2 _enhances nuclear Oct-1 content**. **(A) **InR1-G9 cells were treated with the indicated amount of H_2_O_2 _for 2 or 4 h. Oct-1 expression in whole cell lysates (WCL), nuclear extract (NE), and cytosol were then assessed. **(B, C) **Effect of H_2_O_2 _on Oct-1 nuclear translocation in intestinal GLUTag and pancreatic Ins-1 cell lines, two non-endocrine cell lines (Cos-7 and Caco-2) (**B**) and the primary rat smooth muscle cells (**C**).

Given that oxidative stress in pancreatic islet cells is a major contributor of islet cell damage and subsequent diabetic hyperglycemia, and that Oct-1 is a known sensor for radiation mediated and other types of stress, we assessed whether oxidative stress affects Oct-1 sub-cellular distribution by Western blotting. The InR1-G9 cell line was treated with 100 or 500 μM H_2_O_2 _for 2 or 4 h, and Oct-1 contents in whole cell lysates, as well as in nuclear and cytoplasmic fractions were examined. As shown in Figure 1A, although Oct-1 levels in whole cell lysates were not notably altered by H_2_O_2 _treatment, the content of Oct-1 in nuclei was substantially increased. For cells exposed to 100 μM H_2_O_2 _for 2 h, cytosolic Oct-1 expression was almost undetectable. However, when the exposure time was extended to 4 h, cytosolic Oct-1 expression was restored (Figure 1A). We have also investigated whether oxidative stress would affect Oct-1 nuclear content in other cell lineages. Indeed, H_2_O_2 _treatment enhanced nuclear Oct-1 contents in the intestinal Gcg-expressing GLUTag cell line [13], the pancreatic insulin-expressing Ins-1 cell line, two non-endocrine cell lines, COS-7 and Caco-2 (Figure 1B), as well as primary rat smooth muscle cells (Figure. 1C).

### H_2_O_2 _treatment induces Oct-1 shuttling from cytoplasm into nuclei

To assess the effect of H_2_O_2_/oxidative stress and further examine the effect of cAMP elevation on Oct-1 cytoplasmic-nuclear shuttling, we generated a fusion protein of Oct-1 and enhanced green fluorescence protein (EGFP) (Oct-1-EGFP). The empty EGFP-C3 vector or Oct-1-EGFP construct were transiently transfected into the InR1-G9 cell line. Twenty-four hrs after the transfection, cells were treated with the cAMP promoting agents Forsklin/IBMX (F/I) for 2 h. The expression and sub-cellular compartmentalization of the EGFP tag was visualized using confocal microscopy. A representation result with EGFP empty vector transfection by vehicle and F/I treatment was shown as additional file 1. It appears that EGFP mainly remains in the cytoplasm, regardless of the presence of F/I (Additional File 1A). However, Oct-1-EGFP showed two distinct patterns with regard to fluorescence distribution (Additional File 1B). For Pattern I, fluorescence was observed in a limited cell compartment, which represents Oct-1-EGFP nuclear localization. For Pattern II (after F/I treatment), fluorescence was mainly observed in the cytoplasm, indicating that most of Oct-1 molecules are outside of the nuclei. Additional file 2 top panel shows representative images with Oct-1-EGFP transfected InR1-G9 cells that were treated with F/I, 8-bromo-cAMP and Epac pathway specific cAMP analogue 8-pMeOPT-2'-O-Me-cAMP (Epac). Without a treatment, we observed approximately 58% of Pattern I and 42% of Pattern II of Oct-1 distribution. Following an above treatment for 2 h, significant pattern change was observed. The Pattern I decreased to 17-22% while Pattern II increased to 78-83% (Additional File 2 bottom panel), indicating a stimulated Oct-1 EGFP nuclear exclusion in response to cAMP elevation or Epac activation, which is consistent with our previous observations [12].

**Figure 2:**
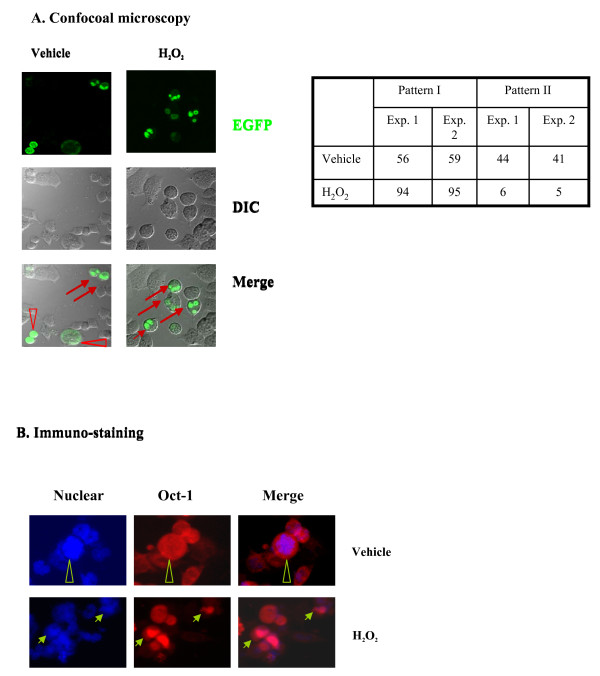
**H_2_O_2 _treatment leads to increased nuclear Oct-1 content**. **(A) **Left panel, InR1-G9 cells were transfected with Oct-1-EGFP and treated with vehicle, or H_2_O_2 _(100 μM) for 1 h before confocal microscopy examination. The right panel shows the counting results. **(B) **InR1-G9 cells were transfected with myc-Oct-1 for 24 h, followed by vehicle or H_2_O_2 _(100 μM) treatment for 1 h. Immunofluorescence staining were conducted, Blue, nuclei, red, myc-Oct-1. Arrow indicates enhanced nuclear myc-Oct-1 or Oct-1-EGFP expression, while triangle indicates mixed expression in both nuclei and cytosol.

We then examined whether H_2_O_2 _treatment affects sub-cellular distributions of Oct-1-EGFP. Oct-1-EGFP was transiently transfected into the InR1-G9 cell line for 24 h, followed by H_2_O_2 _treatment. We found that the treatment of InR1-G9 cells with 100 μM H_2_O_2 _for 0.5, 1, and 2 h led to increased percentages of pattern I cells. Figure 2A (left panel) shows our representative results after a 1 h H_2_O_2 _treatment, cells with nuclear Oct-1-EGFP expression (Pattern I) being sharply increased from 58% to 94% (right panel). We therefore suggest that H_2_O_2 _treatment significantly stimulates nuclear import of Oct-1, which is opposite to the effect induced by cAMP elevation. We then transfected InR1-G9 cells with myc-tagged Oct-1. 24 h after the transfection, cells were treated with vehicle or 100 μM H_2_O_2 _for 1 h before immunofluorescence staining, assessing the distribution of myc-tagged Oct-1. Representative images in Figure 2B indicate that H_2_O_2 _treatment increased nuclear myc-Oct immunofluorescence signal.

### H_2_O_2 _treatment enhances Oct-1 recovery into nuclei

**Figure 3:**
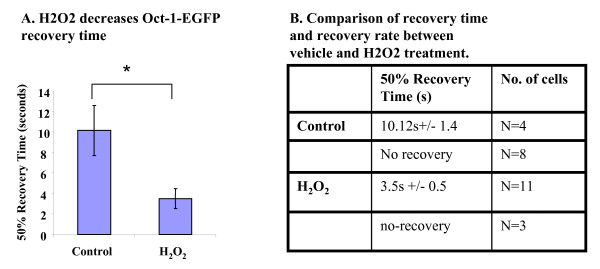
**H_2_O_2 _stimulates OCT-1-EGFP nuclear import**. **(A) **InR1-G9 cells were transfected with Oct-1-EGFP, and treated with vehicle (control), or H_2_O_2 _(100 μM). After photo bleaching of the nuclear fluorescence, the time was recorded for the area to recover to the 50% intensity. (**B) **Comparison of recovery time and recovery rate between vehicle and H_2_O_2 _treated cells.

Recent studies have indicated, with irradiation, DNA-PK activation could lead to enhanced phosphorylation of Oct-1 at its selected Ser/Thr residues [9]. This phosphorylation event may facilitate the binding of Oct-1 to its target gene promoters [9,14,15]. To investigate whether increased percentages of pattern I cells after H_2_O_2 _treatment is a result of increased Oct-1-EGFP nuclear import, we utilized Fluorescence Recovery After Photobleaching (FRAP). For FRAP analysis, the fluorescence molecule in the nuclei is bleached by a laser beam. By recording the time (normally in the ones to tens of seconds range) taken to reach 50% intensity when the fluorescence reaches a new plateau (or termed "50% recovery time") for the same bleached area, one can measure the capability of a molecule to move around over time. The "recovery rate" refers to as the percentage of the recovery observed from total cells bleached. For this study we transfected Oct-1-EGFP into the InR1-G9 cell line. Twenty-four h after the transfection, cells were treated with vehicle or H_2_O_2_. The treated cells were then subjected to the laser photobleaching. The 50% recovery time was recorded as an indicator of Oct-1-EGFP to move from cytoplasm to nuclear. The effect of H_2_O_2 _treatment on Oct-1-EGFP recovery time and the recovery rate by FRAP are shown in Figure. 3A and 3B. As summarized, for cells treated with vehicle, the 50% recovery time was about 10.12 seconds while the recovery rate is only about 33% (4 out of 12). However, for cells received H_2_O_2 _treatment, 50% recovery time was only 3.5 seconds and the recovery rate reached 79% (11 out of 14). These results clearly indicate that H_2_O_2 _treatment stimulates the import of Oct-1 from the cytoplasm into the nuclei, and further suggest that Oct-1 serve as a sensor for oxidative stress.

### H_2_O_2 _treatment activates DNA-PK and JNK

**Figure 4:**
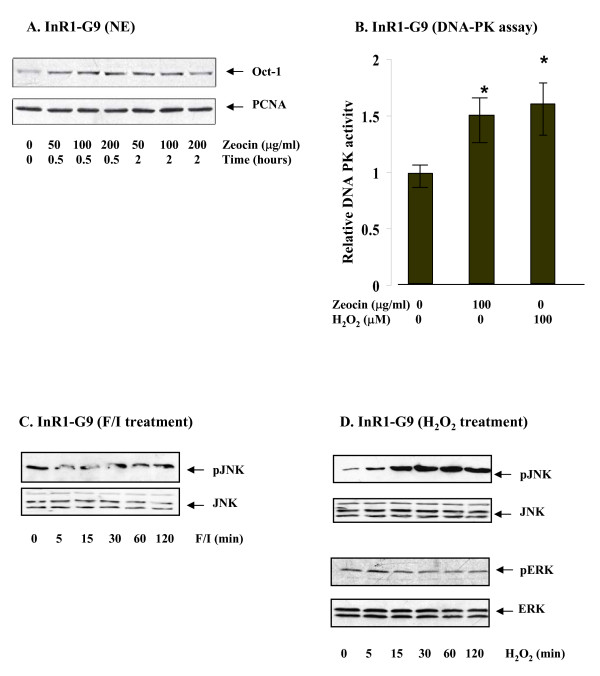
**H_2_O_2 _provoked DNA-PK and JNK activity.(A) **InR1-G9 cells were treated with Zeocin (50, 100 or 200 μg/ml) for indicated time before harvested. Oct-1 expression in the nuclear extract (NE) was then assessed. **(B) **InR1-G9 cells were treated with vehicle, Zeocin (100 μg/ml) or H_2_O_2 _(00 μM) for 2 h before harvested for DNA-PK assay. The values are mean +/- S.E (n = 3). *, P < 0.05. **(C, D) **InR1-G9 cells were treated with forskolin/IBMX (**C**) or H_2_O_2 _(100 μM) (**D**) for indicated time. Whole cell lysates were utilized for assessing the expression of JNK and phosphorylated JNK, ERK and phosphorylated ERK.

Stress induced by irradiation was shown to cause DNA double strand break (DSB) and the activation of DNA-PK, followed by the recruitment of Ku protein complex to the DNA break ends. Zeocine, an antibiotic, was also shown to cause DSB. We then assessed whether oxidative tress induced by H_2_O_2 _treatment leads to increased DNA-PK activity. For this purpose, we have firstly verified that treating InR1-G9 cells with Zeocine, similar to H_2_O_2 _treatment, led to Oct-1 nuclear accumulation (Figure. 4A). This result allowed us to utilize Zeocine as a positive control to assess DNA-PK activity. As shown in Figure. 4B, DNA-PK activity in H_2_O_2 _treated InR1-G9 cells reached to a similar level as that in cells treated with Zeocine. We therefore conclude that in the pancreatic islet cell line InR1-G9, H_2_O_2 _treatment leads to a moderate but significant DNA-PK activation, associated with enhanced Oct-1 nuclear import.

Mitogens, G-protein coupled receptors, and stress were all shown to use complex mitogen activated protein kinase (MAPK) signaling cascades to exert their regulatory functions. Indeed, both cAMP and H_2_O_2 _have been demonstrated to activate MAPK signaling pathways [16-18]. Among the MAPKs, JNK is a known effector of stress induced by genotoxic agents [19]. We have learned that cAMP elevation in InR1-G9 cells led to increased ERK activation, associated with reduced nuclear Oct-1 content [12]. To initiate the examination why cAMP elevation and H_2_O_2 _treatment trigger Oct-1 sub-cellular localization in opposite directions, we examined the effects of these two treatments on ERK and JNK activation in the InR1-G9 cell line. We found that although cAMP elevation induced by F/I treatment, stimulated ERK phosphorylation [12,20], the treatment did not affect JNK phosphorylation (Figure. 4C). H_2_O_2 _treatment, however, generated a stimulatory effect on JNK phosphorylation (Figure. 4D), consistent with our knowledge that JNK signaling is among the effectors of oxidative stress [19,21,22]. The treatment, however, did not affect ERK phosphorylation (Figure. 4D). These observations would further suggest that cAMP signaling and oxidative stress utilize different MAPK in exerting their effects on Oct-1 nuclear-cytoplasmic shuttling, and therefore generate different effects on hormone gene expression.

### Oxidative stress represses Cdx-2 and gcg expression in the InR1-G9 cells

**Figure 5:**
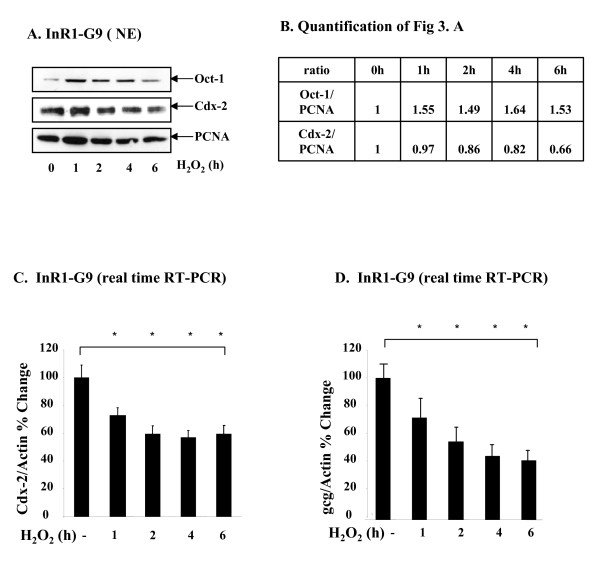
**H_2_O_2 _increased nuclear Oct-1 content, associated with reduced Cdx-2 and gcg expression**. **(A) **InR1-G9 cells were treated with 100 μM H_2_O_2 _for 1 to 6 h, nuclear extract was prepared, followed by the analysis of Oct-1 and Cdx-2 expression by Western blotting. **(B) **The quantification results of Fig 5A. **(C, D) **InR1-G9 cells were treated with 100 μM H_2_O_2 _for 1 to 6 h. Cdx-2 (**C**) and gcg (**D**) mRNA expression was measured by real time RT-PCR.

Since Oct-1 serves as a transcriptional repressor of Cdx-2, expressed in pancreatic and intestinal endocrine cells, we wonder whether increased nuclear content of Oct-1 in response to oxidative stress reduces Cdx-2 expression in such an endocrine cell line. While H_2_O_2 _treatment increased nuclear Oct-1 content in the InR1-G9 cell line (Figure 5A, B), the inhibitory effect on Cdx-2 protein expression, however, was not notable until 6 h (Figure. 5A). This delay could be due to increased stability of Cdx-2 protein in response to a stress [23,24], We therefore directly assessed the effect of H_2_O_2 _treatment on Cdx-2 mRNA expression. As shown in Figure 5C, H_2_O_2 _treatment resulted in about 40% reduction in Cdx-2 mRNA level over the entire 6 h experimental period, confirming that oxidative stress represses Cdx-2 mRNA expression. Additionally, the expression of Gcg mRNA, which is a known downstream target of Cdx-2 in pancreatic α cells [25], is also reduced by H_2_O_2 _treatment, to approximately 50% level (Figure 5D).

## Discussion

An early study by our group indicated that Oct-1 co-transfection stimulated Cdx-2 promoter expression in the pancreatic and intestinal gcg producing cell lines [26]. More recent investigations suggested that Oct-1 could function as a transcriptional repressor [8]. We noticed that cAMP promoting agents, forskolin and IBMX, reduced nuclear Oct-1 content but stimulated Cdx-2 expression. Our further investigations revealed that Oct-1 is able to recruit nuclear co-repressors to Cdx-2 promoter and represses its transcription, while the activation of cAMP-Epac signaling increases Oct-1 nuclear-cytoplasmic shuttling [12]. Thus, Oct-1 serves as a repressor of Cdx-2 and its downstream target Gcg in pancreatic and intestinal endocrine cells [12]. As an abundantly expressed homeobox gene in gut, Cdx-2 plays a critical role in intestinal cell differentiation [23,24,27]. We have shown that it is expressed in intestinal endocrine L cells and stimulates Gcg transcription [25].

It has been shown that *Oct1*^-/- ^cells are more hypersensitive to stress-inducing agents or treatment, such as ionizing radiation [15]. In the present study, we have assessed the effect of H_2_O2 on cytoplasmic-nuclear shuttling of Oct-1. We show in the current study that H_2_O_2 _treatment led to increased Oct-1 nuclear localization by Western blotting, following sub-cellular fractionation; and confocal and fluorescence microcopy, following exogenous expression of EGFP or myc- tagged Oct-1. Increased nuclear shuttling was then directly assessed by FRAP. We found that H_2_O_2 _treatment stimulated the activity of DNA-PK; and that opposite to cAMP elevation, H_2_O_2 _stimulated JNK activity but not ERK activity. Finally, we show that increased nuclear Oct-1 content upon H_2_O_2 _treatment led to approximately 40-50% reduction of Cdx-2 and gcg mRNA expression, which was consistent with our notion that Oct-1 represses Cdx-2 expression. Since DNA-PK activity is responsible for Oct-1 phosphorylation upon irradiation [9], we speculate that increased Oct-1 nuclear shuttling in response to H_2_O_2 _treatment is a result of its phosphorylation by DNA-PK [9]. This, nevertheless, needs to be further investigated.

Intensive investigations have shown that Oct-1 up- or down-regulates the expression of a large profile of target genes in different cell lineages and that this ubiquitously expressed transcription factor is involved in different categories of cellular and molecular activities, from transcriptional regulation to embryonic development [8,28-30]. The role of Oct-1 in mediating metabolic as well as stress/survival signaling pathways, however, was recognized only recently [8,14,15,31]. In addition to the repression of Cdx-2 expression, we have also shown that Oct-1 can repress the expression of the transcription factor carbohydrate responsive element binding protein (ChREBP) [32], which is important in facilitating liver lipogenesis [33]. Insulin, however, stimulated ChREBP transcription. More importantly, the stimulatory effect of insulin was at least partially mediated by attenuating the repressive effect of Oct-1 [32]. These observations collectively suggest that Oct-1 serves as a sensor for metabolic signaling pathways. It represses the expression of important master control genes, such as Cdx-2 in pancreatic islets and ChREBP in hepatocytes. Following the stimulation by a peptide hormone, such as insulin or those that utilize cAMP as the second messenger, Oct-1 is phosphorylated at certain Ser/Thr residues and excluded from the nucleus. This may represent a novel mechanism for peptide hormones in regulating gene expression.

Oct-1 is also known to act as a sensor for stress. Oct-1 deficiency in mice (*Oct-1*^-/-^) is embryonically lethal [29]. Utilizing microarray expression profiling, Tantin *et al. *found that in *Oct-1*^-/- ^fibroblasts a large profile of genes associated with cellular stress exhibited altered expression pattern [15]. Furthermore, Tantin *et al. *[15] and Schild-Poulter *et al. *[34] have shown that in radiation induced stress, Oct-1 could be phosphorylated by DNA-PK at 13 potential Ser/Thr residues within the N terminus of the Oct-1 molecule. Consistent with this finding, *Oct-1*^-/- ^fibroblasts are hypersensitive to γ irradiation, doxorubicin and H_2_O_2 _treatment, and contained elevated level of reactive oxygen species (ROS). Very recently, Kang et al. have demonstrated that Oct-1 is dynamically modulated by phosphorylation *in vivo *following the response to genotoxic and oxidative stress [14]. The stress induced phosphorylated Oct-1 has a higher affinity for DNA binding. Additionally, the interaction between Oct-1 and a distinct group of target promoters is inducible by oxidative stress and these target promoters frequently contain conserved octamer binding sites [14]. We present here that in a battery of cell lines and primary cells, nuclear Oct-1 content is elevated after H_2_O_2 _treatment. We suggest this is due to the result of Oct-1 shuttling from cytoplasm to nuclei, following the phosphorylation by DNA-PK.

Although the activation on DNA-PK by H_2_O_2 _is moderate, the stimulation was comparable with that of Zeocine treatment. Interestingly, Lebrun *et al. *have reported that DNA-PK could phosphorylate another pancreatic islet homeodomain protein PDX-1, and the phosphorylation accelerated PDX-1 proteasome degradation [35]. Therefore, DNA-PK activation in response to oxidative stress may affect pancreatic islet hormone-gene expression through affecting both homeodomain protein expression and degradation. Furthermore, we observed increased JNK phosphorylation in the InR1-G9 cells in response to H_2_O_2 _treatment. Whether this activation is related to Oct-1 nuclear-cytoplamic shuttling deserves a further examination.

Our finding that cAMP elevation stimulates Oct-1 nuclear exclusion and that H_2_O_2 _treatment leads to increased nuclear Oct-1 content place Oct-1 in the centre of signaling cascades that are involved in response to both oxidative stress and hormones/neurotransmitters that utilize cAMP as the second messenger. For this matter, Oct-1 should not be simply considered as a repressor for a cluster of genes. Instead, it is a sensor for both metabolic and stress/survival signaling pathways. Indeed, a recent study shows that Oct-1 mediates the effect of oxidized LDL (oxLDL) in repressing the expression of vascular cytochrome P450 (CYP) monooxygenases [8]. In the coronary arterial endothelial cells, knockdown of Oct-1 expression prevented oxLDL-mediated silencing of CYP expression [8]. Therefore, Oct-1 activation in response to oxidative stress is among the pathological entity in metabolic dysfunction [8], and attenuating the function of Oct-1 improves the dysfunction.

## Conclusion

Based on data presented in this study and elsewhere [8-12], we conclude that the ubiquitously expressed Oct-1 functions to control gene expression in response to cAMP elevation and oxidative stress via a similar nuclear-cytoplasmic shuttling system, which confines Oct-1 to either the nucleus or the cytoplasm.

## Methods

### Reagents, plasmids, cell cultures, and DNA transfection

Forskolin, 3-Isobutyl-1-methylxanthine (IBMX) and Hydrogen peroxide (H_2_O_2_) were purchased from Sigma Aldrich (Oakville, Ontario, Canada). Epac pathway specific cAMP analogue 8-pMeOPT-2'-O-Me-cAMP (ESAC) was provided by BIOLOG Life Sciences Institute (Bremen, Germany). The antibiotics Zeocin was purchased from Invitrogen (Invitrogen Life Technology, Burlington, Ontario, Canada). The plasmid construct Oct-1-EGFP was generated by inserting a copy of human Oct-1 coding sequence [36] into the EGFP-C3 expression vector (Invitrogen Life Technology, Burlington, Ontario, Canada). Hamster pancreatic InR1-G9, mouse large intestinal GLUTag, small intestinal STC-1, Monkey Kidney Fibroblast cell line Cos-7 and the human colon cancer Caco-2 cell lines were maintained as previously described [25,26,37]. The rat pancreatic β cell line Ins-1 and the rat primary islet cell cultures were grown in RPMI medium with 10% fetal bovine serum. The primary smooth muscle cells were isolated from rat aortas and cultured as previously described [38].

### Real time RT-PCR

Complementary DNAs (cDNAs) were generated using a Superscript First-strand RT-PCR kit (Invitrogen Life Technology). Real time RT-PCR was conducted using the QuantiTect SYBR green PCR kit from Qiagen (Mississauga, Ontario, Canada). DNA sequences of the primers utilized for quantitatively assessing mRNA expression by real time RT-PCR are as follows: For hamster Cdx-2: Forward, 5'-CCTAGACAAGGACGTGAGCA-3'; Reverse, 5'-CCTAGACAAGGACGTGAGCA-3'. For hamster gcg: Forward, 5'-AGAAGAAGTCGCCATTGCTG-3'; Reverse, 5'-CGCAGAGATGTTGTCAAGA-3'

### Nuclear and cytosolic protein extraction

The nuclear and cytosolic proteins were extracted based on the method by Schreiber *et al *[39]. Briefly, approximately 1 × 10^6 ^cells collected were washed with phosphate buffered saline (PBS) and pelleted by centrifugation (1500 g for 5 min). The pellet was then re-suspended in 500 μl cold buffer A [10 mM HEPES (pH 8.0), 10 mM KCl, 0.1 mM EDTA, 0.1 mM EGTA, 1 mM DTT and 0.5 mM PMSF] and incubated on ice for 15 min. After the addition of 25 μl of 10% Nonidet NP-40, the cells were vigorously votexed for 10 sec. Following a centrifugation for 30 sec, the supernatant was collected and treated as the cytoplasmic fraction. The nuclear pellet was then resuspended in 60 μl ice-cold buffer C [20 mM HEPES (pH 8.0), 0.4 M NaCl, 1 mM EDTA, 1 mM EGTA, 1 mMDTT, and 1 mM PMSF] and the tube was vigorously rocked at 4°C for 15 min. Nuclear proteins were then collected by a 5 min centrifugation at 4°C.

### Western blotting

The Cdx-2 antibody was generated as previously described [40]. Antibodies against Oct-1 (sc-8024), actin, ERK (sc-94), phosphorylated ERK (sc-7383), JNK (sc-571) phosphorylated JNK (sc-6254), PCNA, and horseradish peroxidase (HRP)-conjugated secondary antibodies were purchased from Santa Cruz Biotechnology, Inc. (Santa Cruz, CA). Preparation of whole cell lysates, and Western blotting were carried out as described previously.

### Confocal microscopy and FRAP analysis

For Confocal microscopy, InR1-G9 cells were transfected with EGFP-C3 or Oct-1-EGFP construct. The cells were treated with Forskolin/IBMX (10 μM each), or 8-bromo-cAMP (100 μM), or the Epac pathway specific cAMP analogue 8pMeOPT-2'-O-ME-cAMP (ESCA, 20 μM), or H_2_O_2 _(100 μM) for the indicated time before visualizing the fluorescence on Zeiss LSM 510 for image capturing. For FRAP analysis, the imaging was carried out on Olympus FV1000 confocal (Olympus, USA). The InR1-G9 cells were grown on the Lab-Tek II chamber plate (Nunc, NY, USA) and transfected with Oct-1-EGFP construct. After 24 hours, the transfected cells were treated with vehicle or 100 μM H_2_O_2 _immediately before imaging. The nuclear region was located and 405 nm diode laser was used for photobleaching. The time for 50% recovery of fluorescence intensity refers to as "50% recovery time", which is the half time between T0 (the moment at bleach) and T1 (when the fluorescence reaches a new plateau, which is normally lower than the original intensity). The "recovery rate" refers to as the percentage of the recovery observed from total number of cells bleached.

### DNA dependent protein kinase assay

The DNA PK assay was performed by using an assay kit from Promega (Promega Corporation, WI), according to manufacturer's instruction. Briefly, Approximately 5 ×10^6 ^InR1-G9 cells treated with either vehicle, Zeocin (100 μg/ml), or H_2_O_2 _(100 μM) were harvested and nuclear extract was prepared. The endogenous DNA from the nuclear extract was removed by DEAE Sepharose column. The DNA PK activity from the nuclear extract was subsequently measured with γ-ATP as a probe, as per manufacturer's instructions.

## Abbreviations

DNA-PK: DNA-dependent protein kinase; EGFP: enhanced green fluorescent protein; Epac: exchange protein directly activated by cAMP; IBMX: 3-isobutyl-1-methylxanthine; JNK: c-jun N-terminal kinase; MEK: mitogen-activatedprotein kinase; OCT: octamer-binding site; Oct-1: octamer transcription factor-1; RT: reverse transcription; H_2_O_2_: hydrogen peroxide.

## Authors' contributions

PW did all the bench work and data analysis. TJ took overall responsibility for the project, and writing up the article. All authors read and approved the final version of the manuscript.

## Supplementary Material

Additional file 1 **Forsklin/IBMX treatment causes Oct-1-EGFP shuttling from nuclear to cytoplasm**. Treating the InR1G9 cell line with forskolin/IBMX led to increased Oct-1-EGFP content in the cytosol. Click here for file

Additional file 2 **cAMP promoting agents cause Oct-1-EGFP shuttling from nuclear to cytoplasm**. Both membrane permeable cAMP analogue and the Epac pathway specific cAMP analogue increased Oct-1-EGFP content in the cytosol. Click here for file

## References

[B1] Accili D Arden KC FoxOs at the crossroads of cellular metabolism, differentiation, and transformation Cell 2004 117 4 421 426 10.1016/S0092-8674(04)00452-0 15137936

[B2] Buteau J Accili D Regulation of pancreatic beta-cell function by the forkhead protein FoxO1 Diabetes Obes Metab 2007 9 Suppl 2 140 146 10.1111/j.1463-1326.2007.00782.x 17919188

[B3] Herr W Sturm RA Clerc RG Corcoran LM Baltimore D Sharp PA Ingraham HA Rosenfeld MG Finney M Ruvkun G The POU domain: a large conserved region in the mammalian pit-1, oct-1, oct-2, and Caenorhabditis elegans unc-86 gene products Genes Dev 1988 2 12A 1513 1516 10.1101/gad.2.12a.1513 3215510

[B4] Ryan AK Blumberg B Rodriguez-Esteban C Yonei-Tamura S Tamura K Tsukui T de la Pena J Sabbagh W Greenwald J Choe S Pitx2 determines left-right asymmetry of internal organs in vertebrates Nature 1998 394 6693 545 551 10.1038/29004 9707115

[B5] Wysocka J Herr W The herpes simplex virus VP16-induced complex: the makings of a regulatory switch Trends Biochem Sci 2003 28 6 294 304 10.1016/S0968-0004(03)00088-4 12826401

[B6] Chandran UR Warren BS Baumann CT Hager GL DeFranco DB The glucocorticoid receptor is tethered to DNA-bound Oct-1 at the mouse gonadotropin-releasing hormone distal negative glucocorticoid response element J Biol Chem 1999 274 4 2372 2378 10.1074/jbc.274.4.2372 9891005

[B7] Chandran UR Attardi B Friedman R Zheng Z Roberts JL DeFranco DB Glucocorticoid repression of the mouse gonadotropin-releasing hormone gene is mediated by promoter elements that are recognized by heteromeric complexes containing glucocorticoid receptor J Biol Chem 1996 271 34 20412 20420 10.1074/jbc.271.34.20412 8702778

[B8] Thum T Borlak J LOX-1 receptor blockade abrogates oxLDL-induced oxidative DNA damage and prevents activation of the transcriptional repressor Oct-1 in human coronary arterial endothelium J Biol Chem 2008 283 28 19456 19464 10.1074/jbc.M708309200 18390905

[B9] Schild-Poulter C Shih A Tantin D Yarymowich NC Soubeyrand S Sharp PA Hache RJ DNA-PK phosphorylation sites on Oct-1 promote cell survival following DNA damage Oncogene 2007 26 27 3980 3988 10.1038/sj.onc.1210165 17213819

[B10] Schild-Poulter C Shih A Yarymowich NC Hache RJ Down-regulation of histone H2B by DNA-dependent protein kinase in response to DNA damage through modulation of octamer transcription factor 1 Cancer Res 2003 63 21 7197 7205 14612514

[B11] Schild-Poulter C Su A Shih A Kelly OP Fritzler MJ Goldstein R Hache RJ Association of autoantibodies with Ku and DNA repair proteins in connective tissue diseases Rheumatology (Oxford) 2008 47 2 165 171 10.1093/rheumatology/kem338 18208821

[B12] Wang P Wang Q Sun J Wu J Li H Zhang N Huang Y Su B Li RK Liu L POU homeodomain protein Oct-1 functions as a sensor for cyclic AMP J Biol Chem 2009 284 39 26456 26465 10.1074/jbc.M109.030668 19617623 PMC2785334

[B13] Drucker DJ Jin T Asa SL Young TA Brubaker PL Activation of proglucagon gene transcription by protein kinase-A in a novel mouse enteroendocrine cell line Mol Endocrinol 1994 8 12 1646 1655 10.1210/me.8.12.1646 7535893

[B14] Kang J Gemberling M Nakamura M Whitby FG Handa H Fairbrother WG Tantin D A general mechanism for transcription regulation by Oct1 and Oct4 in response to genotoxic and oxidative stress Genes Dev 2009 23 2 208 222 10.1101/gad.1750709 19171782 PMC2648538

[B15] Tantin D Schild-Poulter C Wang V Hache RJ Sharp PA The octamer binding transcription factor Oct-1 is a stress sensor Cancer Res 2005 65 23 10750 10758 10.1158/0008-5472.CAN-05-2399 16322220

[B16] Zhang Y Chen F Reactive oxygen species (ROS), troublemakers between nuclear factor-kappaB (NF-kappaB) and c-Jun NH(2)-terminal kinase (JNK) Cancer Res 2004 64 6 1902 1905 10.1158/0008-5472.CAN-03-3361 15026320

[B17] Shen HM Pervaiz S TNF receptor superfamily-induced cell death: redox-dependent execution Faseb J 2006 20 10 1589 1598 10.1096/fj.05-5603rev 16873882

[B18] Dumaz N Marais R Integrating signals between cAMP and the RAS/RAF/MEK/ERK signalling pathways. Based on the anniversary prize of the Gesellschaft fur Biochemie und Molekularbiologie Lecture delivered on 5 July 2003 at the Special FEBS Meeting in Brussels Febs J 2005 272 14 3491 3504 10.1111/j.1742-4658.2005.04763.x 16008550

[B19] Fritz G Kaina B Late activation of stress kinases (SAPK/JNK) by genotoxins requires the DNA repair proteins DNA-PKcs and CSB Mol Biol Cell 2006 17 2 851 861 10.1091/mbc.E05-07-0606 16319174 PMC1356594

[B20] Chen L Wang P Andrade CF Zhao IY Dube PE Brubaker PL Liu M Jin T PKA independent and cell type specific activation of the expression of caudal homeobox gene Cdx-2 by cyclic AMP Febs J 2005 272 11 2746 2759 10.1111/j.1742-4658.2005.04694.x 15943809

[B21] Hotamisligil GS Role of endoplasmic reticulum stress and c-Jun NH2-terminal kinase pathways in inflammation and origin of obesity and diabetes Diabetes 2005 54 Suppl 2 S73 78 10.2337/diabetes.54.suppl_2.S73 16306344

[B22] Temkin V Karin M From death receptor to reactive oxygen species and c-Jun N-terminal protein kinase: the receptor-interacting protein 1 odyssey Immunol Rev 2007 220 8 21 10.1111/j.1600-065X.2007.00560.x 17979836

[B23] Boulanger J Vezina A Mongrain S Boudreau F Perreault N Auclair BA Laine J Asselin C Rivard N Cdk2-dependent phosphorylation of homeobox transcription factor CDX2 regulates its nuclear translocation and proteasome-mediated degradation in human intestinal epithelial cells J Biol Chem 2005 280 18 18095 18107 10.1074/jbc.M502184200 15741163

[B24] Gross I Lhermitte B Domon-Dell C Duluc I Martin E Gaiddon C Kedinger M Freund JN Phosphorylation of the homeotic tumor suppressor Cdx2 mediates its ubiquitin-dependent proteasome degradation Oncogene 2005 24 54 7955 7963 10.1038/sj.onc.1208945 16027724

[B25] Jin T Drucker DJ Activation of proglucagon gene transcription through a novel promoter element by the caudal-related homeodomain protein cdx-2/3 Mol Cell Biol 1996 16 1 19 28 8524295 10.1128/mcb.16.1.19 PMC230974

[B26] Jin T Li H Pou homeodomain protein OCT1 is implicated in the expression of the caudal-related homeobox gene Cdx-2 J Biol Chem 2001 276 18 14752 14758 10.1074/jbc.M008277200 11278400

[B27] Beck F The role of Cdx genes in the mammalian gut Gut 2004 53 10 1394 1396 10.1136/gut.2003.038240 15361482 PMC1774238

[B28] Vazquez-Martinez R Leclerc GM Wierman ME Boockfor FR Episodic activation of the rat GnRH promoter: role of the homeoprotein oct-1 Mol Endocrinol 2002 16 9 2093 2100 10.1210/me.2002-0139 12198245

[B29] Wang VE Schmidt T Chen J Sharp PA Tantin D Embryonic lethality, decreased erythropoiesis, and defective octamer-dependent promoter activation in Oct-1-deficient mice Mol Cell Biol 2004 24 3 1022 1032 10.1128/MCB.24.3.1022-1032.2004 14729950 PMC321444

[B30] Kiyota T Kato A Altmann CR Kato Y The POU homeobox protein Oct-1 regulates radial glia formation downstream of Notch signaling Dev Biol 2008 315 2 579 592 10.1016/j.ydbio.2007.12.013 18241856

[B31] Shakya A Cooksey R Cox JE Wang V McClain DA Tantin D Oct1 loss of function induces a coordinate metabolic shift that opposes tumorigenicity Nat Cell Biol 2009 11 3 320 327 10.1038/ncb1840 19219035

[B32] Sirek AS Liu L Naples M Adeli K Ng DS Jin T Insulin stimulates the expression of carbohydrate response element binding protein (ChREBP) by attenuating the repressive effect of Pit-1, Oct-1/Oct-2, and Unc-86 homeodomain protein octamer transcription factor-1 Endocrinology 2009 150 8 3483 3492 10.1210/en.2008-1702 19359385

[B33] Uyeda K Repa JJ Carbohydrate response element binding protein, ChREBP, a transcription factor coupling hepatic glucose utilization and lipid synthesis Cell Metab 2006 4 2 107 110 10.1016/j.cmet.2006.06.008 16890538

[B34] Schild-Poulter C Shih A Tantin D Yarymowich NC Soubeyrand S Sharp PA Hache RJ DNA-PK phosphorylation sites on Oct-1 promote cell survival following DNA damage Oncogene 2007 17213819 10.1038/sj.onc.1210165

[B35] Lebrun P Montminy MR Van Obberghen E Regulation of the pancreatic duodenal homeobox-1 protein by DNA-dependent protein kinase J Biol Chem 2005 280 46 38203 38210 10.1074/jbc.M504842200 16166097

[B36] Tanaka M Herr W Differential transcriptional activation by Oct-1 and Oct-2: interdependent activation domains induce Oct-2 phosphorylation Cell 1990 60 3 375 386 10.1016/0092-8674(90)90589-7 2302733

[B37] Xu F Li H Jin T Cell type-specific autoregulation of the Caudal-related homeobox gene Cdx-2/3 J Biol Chem 1999 274 48 34310 34316 10.1074/jbc.274.48.34310 10567407

[B38] Matsubayashi K Fedak PW Mickle DA Weisel RD Ozawa T Li RK Improved left ventricular aneurysm repair with bioengineered vascular smooth muscle grafts Circulation 2003 108 Suppl 1 II219 225 12970236 10.1161/01.cir.0000087450.34497.9a

[B39] Schreiber E Matthias P Muller MM Schaffner W Rapid detection of octamer binding proteins with 'mini-extracts', prepared from a small number of cells Nucleic Acids Res 1989 17 15 6419. 10.1093/nar/17.15.6419 2771659 PMC318318

[B40] Trinh KY Jin T Drucker DJ Identification of domains mediating transcriptional activation and cytoplasmic export in the caudal homeobox protein Cdx-3 J Biol Chem 1999 274 9 6011 6019 10.1074/jbc.274.9.6011 10026228

