# Supplementary material for: Hydrogen peroxide stimulates nuclear import of the POU homeodomain protein Oct-1 and its repressive effect on the expression of Cdx-2
Source: BMC Cell Biol. 2010 Jul 16;11:56. doi: 10.1186/1471-2121-11-56 (PMC2913919; doi:10.1186/1471-2121-11-56)
Supplement: Additional file 1 — Forsklin/IBMX treatment causes Oct-1-EGFP shuttling from nuclear to cytoplasm. Treating the InR1G9 cell line with forskolin/IBMX led to increased Oct-1-EGFP content in the cytosol. [file 1471-2121-11-56-S1.PDF]

Additional Files

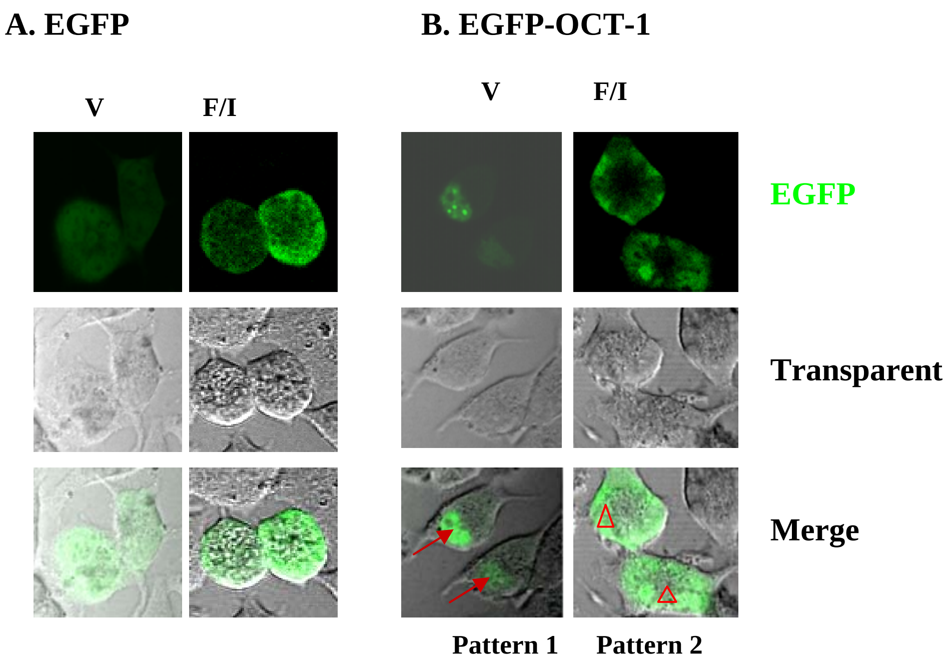

Additional File 1. Forsklin/IBMX treatment causes Oct-1-EGFP shuttling from nuclear to cytoplasm. (A) InR1-G9 cells were transfected with EGFP and treated with vehicle (V), or forskolin/IBMX (10 mM each, F/I). **(B)** InR1-G9 cells were transfected with Oct-1-EGFP and treated with vehicle (V), or forskolin/IBMX (10 mM each, F/I).
