# Supplementary material for: Hydrogen peroxide stimulates nuclear import of the POU homeodomain protein Oct-1 and its repressive effect on the expression of Cdx-2
Source: BMC Cell Biol. 2010 Jul 16;11:56. doi: 10.1186/1471-2121-11-56 (PMC2913919; doi:10.1186/1471-2121-11-56)
Supplement: Additional file 2 — cAMP promoting agents cause Oct-1-EGFP shuttling from nuclear to cytoplasm. Both membrane permeable cAMP analogue and the Epac pathway specific cAMP analogue increased Oct-1-EGFP content in the cytosol. [file 1471-2121-11-56-S2.PDF]

Supplementary Figure S2.

A. EGFP-Oct-1

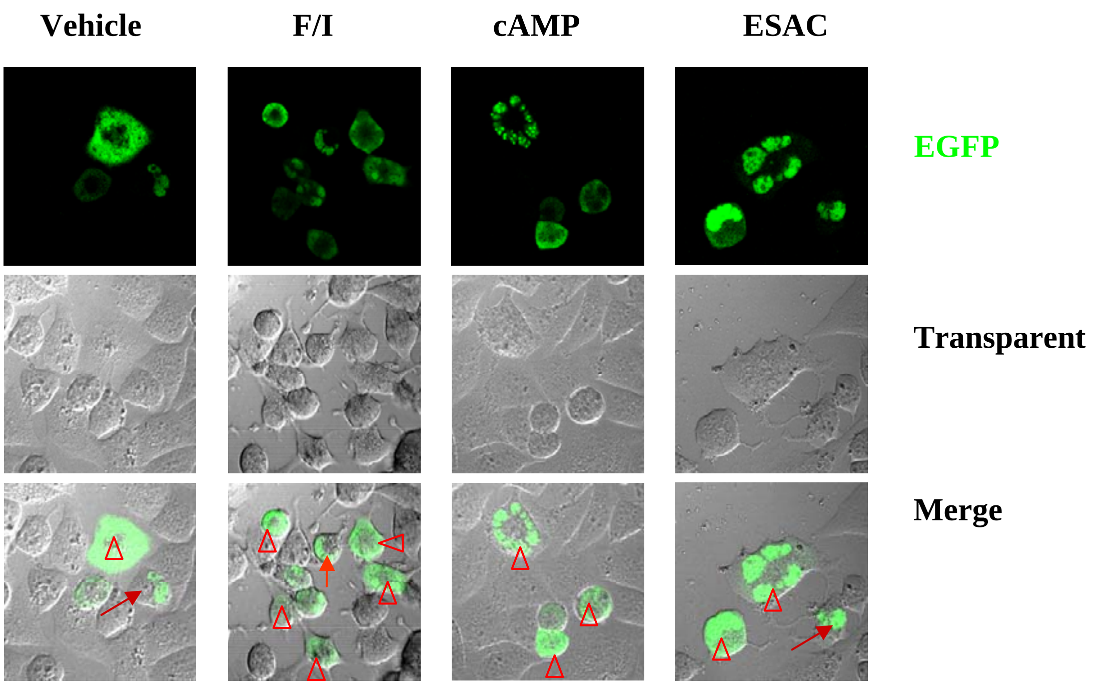

B.

	Pattern I		Pattern II	
	Exp. 1	Exp. 2	Exp. 1	Exp. 2
Vehicle	58	56	42	44
F/I	17	19	83	81
cAMP	19	23	81	77
Epac	22	15	78	85

Additional File 2. cAMP promoting agents cause Oct-1-EGFP shuttling from nuclear to cytoplasm. (A) InR1-G9 cells were transfected with EGFP and treated with vehicle (V), or forskolin/IBMX (10 μM each, F/I), 8-bromo-cAMP (100 μM) and ESAC (8-pMeOPT-2'-O-Me-cAMP, 20 μM). **(B)** The counting results from Panel A.
